# Estimation of Heritability under Correlated Errors Using the Full-Sib Model

**DOI:** 10.3390/genes14040788

**Published:** 2023-03-24

**Authors:** Amrit Kumar Paul, Himadri Shekhar Roy, Ranjit Kumar Paul, Prakash Kumar, Md Yeasin

**Affiliations:** ICAR-Indian Agricultural Statistics Research Institute, New Delhi 110012, India

**Keywords:** autoregressive, AR(1), AR(2), heritability, MSE, two-way classification

## Abstract

In plant and animal breeding, sometimes observations are not independently distributed. There may exist a correlated relationship between the observations. In the presence of highly correlated observations, the classical premise of independence between observations is violated. Plant and animal breeders are particularly interested to study the genetic components for different important traits. In general, for estimating heritability, a random component in the model must adhere to specific assumptions, such as random components, including errors, having a normal distribution, and being identically independently distributed. However, in many real-world situations, all of the assumptions are not fulfilled. In this study, correlated error structures are considered errors that are associated to estimate heritability for the full-sib model. The number of immediately preceding observations in an autoregressive series that are used to predict the value at the current observation is defined as the order of the autoregressive models. First-order and second-order autoregressive models i.e., AR(1) and AR(2) error structures, have been considered. In the case of the full-sib model, theoretical derivation of Expected Mean sum square (EMS) considering AR(1) structure has been obtained. A numerical explanation is provided for the derived EMS considering AR(1) structure. The predicted mean squares error (MSE) is obtained after including the AR(1) error structures in the model, and heritability is estimated using the resulting equations. It is noticed that correlated errors have a major influence on heritability estimation. Different correlation patterns, such as AR(1) and AR(2), can be inferred to change heritability estimates and MSE values. To attain better results, several combinations are offered for various scenarios.

## 1. Introduction

Genetic improvement in plants and animals is primarily determined by the degree to which desirable characteristics are inherited and depends on the accuracy of selection and selection intensity, as well as on the amount of genetic variation, etc. [1]. The availability of genetic diversity, the link between numerous yield and yield-related variables, and heritability are critical for identifying potential genotypes for the development of crop varieties and animal breeds. In real-world phenomena in plant and animal breeding, it has been observed that observations are not independently distributed and that some form of connection exists between the observations [2]. The classical premise of independence among observations is violated in the presence of highly correlated patterns in the data. Plant and animal breeders are particularly interested in knowledge of the genetic components of variations in crucial characteristics [3]. Therefore, from the perspective of plant and animal breeding programs, it is crucial to estimate different genetic variations and make assumptions about their inheritance based on estimates of different genetic characteristics [2]. To tackle the requirements and to improve the crop and animal breeding programs, extensive research on statistical methods for genetic advancement is required.

In statistical modelling aspects, prediction of phenotypic variability is influenced by several factors [2]. In some cases, the general linear model suffers due to violations of the assumptions, and it is fascinating to investigate the impact of correlated error components besides correlated observations to arrive at a random error component. As a result, there is a need to investigate a model in which structural variation caused by error may be further considered for a random error model. In some real-world scenarios, response variables do not adhere to the normality assumptions, Roy et al. [3] implemented a Bayesian linear mixed model for estimation of heritability using pedigree data. It also encourages the use and development of statistical techniques that allow the amount of variability, due to different causes at both the genetic and phenotypic levels, to be assessed scientifically, and allows factors to be compared with a relatively high degree of accuracy, and breeding values to be predicted more efficiently. To date, a significant range of statistical methods for analyzing random and fixed effects models are available in the literature. The development of methods for estimating variance components began a long time ago, in the early twentieth century. Fisher [4] made a significant addition to variance component models by proposing the analysis of the variance technique of estimation. Cochran [5] pioneered the use of unbalanced data. Henderson [6] described a method for determining variance components in a challenging scenario using unbalanced data. Because of the shortcomings (negativity, lack of distributional features) of ANOVA (Analysis of Variance) estimators, other techniques such as ML (Maximum Likelihood), REML (Restricted Maximum Likelihood), MINQUE (Minimum Norm Quadratic Unbiased Estimation), and others evolved.

Again, numerous approaches are available in the literature for assessing the correlation between observations. Durbin and Watson [7] provided a method for determining the presence of a first-order autocorrelation disturbance in the error term. Diblasi and Bowman [8] provided a test statistic and graphical approach for assessing evidence for the presence of a spatial correlation in data. In case of half-sib data, Singh et al. [9] estimate variance components by considering correlated errors which follow autoregressive of order one i.e., AR(I). Costa et al. [10] estimate genetic factors of test day record for fat and protein yields using autoregressive multiple lactation animal models. The term autoregression refers to a regression of a variable against itself. In time series concept, autoregression is a value that is regressed on previous values from the same time series. The order of an autoregressive series is defined as the number of immediately preceding observations in the series that are used to predict the value at the current observation. Under this study, we have used first-order and second-order autoregressive models as AR(1) and AR(2), respectively. Orunmuyi et al. [11] used the maternal half-sib (dam variance) and full-sib (sire + dam variance) components to assess the genetic parameters of fertility and hatchability in two strains of Rhode Island Red (RIR) chickens, denoted as Strain A and Strain B, respectively. Rameez et al. [12] studied to evaluate the performance of Magur (*Clarias magur*) raised in a two-year class, estimate their heritabilities at stocking and harvest, as well as determine the genetic and phenotypic correlations between them. Using information from incredibly small family samples, Ødegård and Meuwissen [13] explored how sharing of identities by descent across the entire genome among close relatives can be utilized to quantify additive genetic diversity originating entirely from within-family variation. It was assumed that information from genome-wide markers could be used to accurately recreate genomic identity-by-descent relationships when estimating genetic variation from phenotypic data. The outcomes were contrasted with those of conventional pedigree-based genetic analysis. Estaghvirou et al. [14] explored how outliers affect the accuracy and robustness of genomic prediction systems in plant breeding. Lourenço et al. [15] explored the robust estimate of heritability and prediction accuracy in plant breeding, using simulation and empirical data. Hodge and Acosta [16] proposed an algorithm for full-sib genetic dataset analysis with extended mixed-model software. The variance component estimates, genetic parameter estimates, and BLUP solutions for genetic values provided by the method are essentially the same as those created by specialized genetic software programs. The method for determining the theoretical probability of heritability (*h*^2^) estimations from full-sib analysis exceeding unity was described by Prabhakaran and Sharma [17]. Two hundred and fifty six full-sib families of maize (*Zea mays* L.) were examined by Marker and Joshi [18] at two distinct degrees of fertility. It was discovered that the additive genetic variance was more significant for grain yield per plant than the variance resulting from dominance deviations. Keeping the impact of the correlated error structure in mind, the development of a statistical technique for estimating genetic parameters when errors are correlated is an important research topic in the field of statistical genetics. The correlation contained in the error structure is overlooked in the standard analytic technique of field experimental data. We cannot ignore the correlation impact when there are highly associated patterns in the data. As a result, it becomes vital to seek out these instances and ways of investigation. Because theories for estimating variance components are only accessible in the literature for uncorrelated errors, the current study was undertaken with the goal in mind to investigate the effect of correlated errors on the quantitative trait inheritance in the case of a full-sib model.

This paper is organized as follows. Section 2 describes the statistical approach to estimating the heritability under a two-way nested model. Derivation of MSE for a full-sib model under correlated errors considering AR(1) structure is shown. Section 3 discusses the results and includes a discussion of the proposed methodology on a simulated dataset, and compared with ANOVA (Analysis of Variance) estimators, other techniques, such as ML (Maximum Likelihood), REML (Restricted Maximum Likelihood), and MINQUE (Minimum Norm Quadratic Unbiased Estimation). The paper is concluded with a Conclusion section.

## 2. Materials and Methods

One of the primary needs for studying the statistical characteristics of genetic parameters was to simulate statistical-biological models with known population parameters. In general, different procedures can be used to estimate heritability, among them, those based on offspring regression on parents and on sib correlation or sib analysis have several advantages and disadvantages. The accuracy and bias of the estimators are determined by the relationship between them, used in the study. At present, we are solely interested in using sib analysis to estimate heritability. As stated below, data generation for correlated and uncorrelated instances is performed using a two-way classification model. In this work, Roningen [19] employed simulation models, specifically two-way nested models (full-Sib’s model) for estimating heritability. The following is a brief overview of the Monte Carlo method:

### 2.1. Two-Way Nested Model

This is commonly referred to as the full-sib analysis model and may be expressed as

$$Y_{ijk}=\mu +s_{i}+d_{ij}+e_{ijk}$$

where,

$s_{i}=$ the effect of *i*th sire

$d_{ij}=$ the effect of *j*th dam mated to *i*th sire

$e_{ijk}=$ the random effect associated with *k*th member of the *ij*th full-sib group.

The simulation model used for generating full-sib data is given as follows:

$$Y_{ijk}=\mu +\sigma_{s}a_{i}+\sigma_{d}a_{ij}+\sigma_{e}a_{ijk}$$

where *µ* is considered as a general mean and $\sigma_{s}$, $\sigma_{d}$, $\sigma_{e}$ represents the standard deviation of the sire component, dam component, and the error component, respectively, and $a_{i}$, $a_{ij}\;$ and $a_{ijk}\;$ are standard normal variates.

### 2.2. Estimation of Heritability by Full-Sib Correlation

Let, $t^{\prime }$ be the estimated intra-class correlation coefficient between full-sib, then an estimate of heritability can be derived as correlation (Falconer [2]).

$${\hat{h}}^{2}=2t^{\prime }$$

The genetic composition of this estimate of heritability is

$$\frac{\left[V_{A}+\frac{1}{2}V_{D}+\frac{1}{2}V_{AA}+\frac{1}{4}V_{AD}+\frac{1}{8}V_{DD}+\cdots \right]}{V_{P}}$$

Here, *V_A_*, *V_D_*, *V_AA_*, *V_AD_*, and *V_DD_* are defined as variance due to additive effect, variance due to dominance effect, variance due to interaction of additive components, variance due to interaction of additive and dominance effect, and variance due to interaction of dominance component, respectively. Thus, this estimate is subject to bias from dominance deviations, as well as from non-allelic interactions. The estimate of heritability is the least reliable of all the estimates of heritability.

The statistical model for estimation of heritability based on the full-sib mating design with *n* offspring per dam is

$$Y_{ijk}=\mu +s_{i}+d_{ij}+e_{ijk}$$

where $Y_{ijk}$ is the measurement of a character on the *k*th progeny of the *j*th dam mated to the *i*th sire. μ is the general mean; *s_i_* is the sire effect common to all the progeny of the *i*th sire; *d_ij_* is the dam’s effect common to all the progeny of the *j*th dam mated to the *i*th sire and *e_ijk_* is random deviation.

All effects except *μ* are random and independent, with expectations of zero and variances

$$E\left(s_{i}^{2}\right)=\sigma_{s}^{2};E\left(d_{ij}^{2}\right)=\sigma_{d}^{2};\;and\;E\left(e_{ijk}^{2}\right)=\sigma_{e}^{2}$$

The data on progeny are then subjected to hierarchical analysis of variance; between sires, between dams within sires, and within dams and within sires to get the estimates of sire and dam components of variance. The form of analysis along with mean squares and their composition in terms of the observational components of variance are shown in Table 1.

Now, we know that

$$\sigma_{p}^{2}=\sigma_{y}^{2}=\sigma_{s}^{2}+\sigma_{d}^{2}+\sigma_{e}^{2}$$

$$\sigma_{s}^{2}+\sigma_{d}^{2}=COV\left(FS\right)$$

$$\sigma_{d}^{2}=COV\left(FS\right)-COV\left(HS\right)$$

$$and\;\sigma_{s}^{2}=COV\left(HS\right)$$

Noting the genetic composition of the variance components $\sigma_{s}^{2}+\sigma_{d}^{2}\;\mathrm{and}\;\sigma_{e}^{2}$, three different estimates of heritability can be obtained from these variance components and are as follow:

Sire components:

$${\hat{h}}_{s}^{2}=\frac{4{\hat{\sigma }}_{s}^{2}}{{\hat{\sigma }}_{s}^{2}+{\hat{\sigma }}_{d}^{2}+{\hat{\sigma }}_{e}^{2}}$$

Dam component:

$${\hat{h}}_{d}^{2}=\frac{4{\hat{\sigma }}_{d}^{2}}{{\hat{\sigma }}_{s}^{2}+{\hat{\sigma }}_{d}^{2}+{\hat{\sigma }}_{e}^{2}}$$

Sire + Dam component:

$${\hat{h}}_{\left(s+d\right)}^{2}=\frac{2\left({\hat{\sigma }}_{s}^{2}+{\hat{\sigma }}_{d}^{2}\right)}{{\hat{\sigma }}_{s}^{2}+{\hat{\sigma }}_{d}^{2}+{\hat{\sigma }}_{e}^{2}}=2t^{\prime }$$

The estimate of heritability by full-sib correlation was obtained by taking the average of sire and dam components and its sampling variance was determined by the approximate formula of sampling variance of an intra-class correlation coefficient.

### 2.3. Correlated Case

Suppose that sires are independent but within sire, progenies are correlated. Further, assume that the correlated errors follow AR(1) i.e.,

$$e_{ij}=\rho e_{i}\left(j-1\right)+\eta_{ij},$$

where, $\eta_{ij}$ is considered as random error component, $|\rho |<1,\mathrm{Var}\left(\eta_{ij}\right)=\frac{\sigma_{e}^{2}}{1-\rho^{2}}$ and $\eta_{ij}\sim IIDN\left(0.1\right)\;for\;j>1.$

From the above equation, $e_{ij}$ s are generated.

In case of AR(2),

$$e_{ij}=\rho_{1}e_{i\left(j-1\right)}+\rho_{2}e_{i\left(j-2\right)}+\eta_{ij}$$

where, $\eta_{ij}$ is considered as random error component.

In a similar fashion, we have generated the correlated data for different error structures other than AR(1) e.g., AR(2), a function of a distance, etc.

### 2.4. Derivation of MSE for Full-Sib Model under Correlated Errors (AR(1))

The usual full-sib model is as follows:

$$Y_{ijk}=\mu +s_{i}+d_{ij}+e_{ijk}$$

here,

*μ* = Effect common to all individuals

*s_i_* = *i*th sire effect $E\left(s_{i}\right)=0,\;V\left(s_{i}\right)=\sigma_{s}^{2}$

*d_ij_* = Effect due to *j*th dam mated to the *i*th sire

$$E\left(d_{i}\right)=0,\;V\left(d_{ij}\right)=\sigma_{d}^{2}$$

*i* = 1, 2, … *S*→$E\left(s_{i}^{2}\right)=\sigma_{s}^{2}$

*j* = 1, 2, … *n_i_*→$E\left(d_{ij}\right)=\sigma_{d}^{2}$

*k* = 1, 2, … *n_ij_*→ $\;E\left(e_{ijk}^{2}\right)=\sigma_{e}^{2}$

$$e_{ijk}=\rho e_{ij\left(k-1\right)}+\eta_{ijk}$$

where $\left|\rho \right|<1$, $Var\left(\eta_{ijk}\right)=\frac{\sigma_{e}^{2}}{1-\rho^{2}}$ and $\eta_{ijk}\approx IIDN\left(0,1\right)for\;k>1.$

Assumptions:

$$E(e_{ijk})=0,$$

*E*(*s*) = 0

$$E\left(e_{ijk}^{2}\right)=\sigma_{e}^{2}$$

$$E\left(s_{i}^{2}\right)=\sigma_{s}^{2}$$

$$Cov\left(s_{i},s_{i^{\prime }}\right)=0\;,\;\forall \;i=i^{\prime }$$

$$Cov\left(s_{i},se_{i^{\prime }jk}\right)=0\;,\;\forall \;i=i^{\prime }$$

$$Cov\left(s_{i},e_{i^{\prime }jk}\right)=0\;,\;\forall \;i\;and\;i^{\prime }$$

$$Cov\left(e_{ijk},e_{i^{\prime }jk}\right)=0\;,\;\forall \;i\;and\;i^{\prime }$$

$$Cov\left(e_{ijk},{e_{ijk}}^{\prime }\right)=\rho^{\left|k-k^{\prime }\right|}\sigma_{e}^{2}\;,\;\forall \;k\;and\;k^{\prime }$$

From the first principal, we have obtained the following equation for the above model.

$$E\left(MSA\right)=Q\sigma_{e}^{2}+\frac{1}{s-1}\left[\underset{i}{\sum }\underset{j}{\sum }\frac{n_{ij}^{2}}{n_{i.}}-\underset{i}{\sum }\underset{j}{\sum }\frac{n_{ij}^{2}}{N}\right]\sigma_{s}^{2}$$

$$+\frac{1}{s-1}\left[N-\frac{1}{N}\underset{i}{\sum }n_{i.}^{2}\right]\sigma_{d}^{2}$$

$$E\left(MSB\right)=Q\sigma_{e}^{2}+\frac{1}{\sum (n_{i.}-1)}\left[N-\underset{i}{\sum }\left(\underset{j}{\sum }\frac{n_{ij}^{2}}{n_{i}.}\right)\right]\sigma_{d}^{2}$$

$$E\left(MSE\right)=Q\sigma_{e}^{2}$$

where, $Q=\left(1+\rho +\rho^{2}+\ldots +\rho^{n_{ij-1}}\right)$.

$$E\left(MSA\right)=Q\sigma_{e}^{2}+\lambda_{2}\sigma_{s}^{2}+\lambda_{3}\sigma_{d}^{2}$$

$$E\left(MSB\right)=Q\sigma_{e}^{2}+\lambda_{1}\sigma_{d}^{2}$$

$$E\left(MSE\right)=Q\sigma_{e}^{2}$$

## 3. Result and Discussion

### 3.1. Estimation of Heritability and MSE Values in Case of Correlated Errors (AR(1)) and Different Sample Sizes for the Different Parametric Values of Heritability

The data were generated from a population with low and high heritability for various sample sizes and family structures. Data have been generated using different heritability values i.e., high, and low (0.5, 0.1), using a full-sib model and different sample sizes 100 and 500 and different correlations of errors A(1) and AR(2). ρ = −1 to +1. After generating the data, variance components are estimated using SAS Proc Varcomp. ANOVA, ML, REML, and MIVQUE methods are used. The heritability estimates, along with MSE (Means Square Error), are obtained and given in Table 2 and Table 3. In almost all cases, biased estimates are obtained. Estimates for considering only sire components are better than considering both sire and dam components and dam components alone.

### 3.2. Estimation of Heritability and MSE Values in Case of Correlated Errors (AR(2)) and Different Sample Sizes for the Different Parametric Values of Heritability

The data were generated from a population with low and high heritability for various sample sizes and family structures. The heritability estimates, along with MSE (Means Square Error), are obtained and shown in Table 4 and Table 5. It is noticed that in almost all cases, biased estimates are obtained. Estimates for considering only sire components are better than considering sire and dam components and dam components alone. A combination of correlation i.e., (0, −0.5) and (0, 0.5) provide better results than any other combination. Increasing sample size decreases the MSE values.

### 3.3. Estimation of Heritability and MSE Values in Case of Correlated Errors (AR(1)) and Different Sample Sizes and Different Parametric Values Heritability Using Derived Formulae

The heritability estimates along with MSE (Means Square Error) are obtained and given in Table 6. The expected mean sum of squares due to error is overestimated when the correlation is negative, and they increase as the degree of correlation increases. However, these expected mean sums of a square are underestimated if errors are positively correlated and they decrease with an increase in the degree of correlation and approach to 0 as ρ tends to unity. On the other hand, only reverse results are obtained for estimating the mean sums of squares due to sire i.e., the expected mean sums of squares are under-estimated when ρ is negative and they are overestimated if the correlation is positive. As ρ tends to unity, the expected mean sum of squares due to sire approaches its maximum value. Heritability values are overestimated if the correlation is positive. The same trend follows for all levels of heritability. Also, heritability increases from zero to nearly four as the autoregressive value increases.

.

In the present work, a full-sib model is used to generate data. AR(1) and AR(2) errors are employed in this case. In the case of a full-sib model, equations for E(MSE) and heritability estimation in the presence of AR(1) correlation in the error are derived. Different AR(1) values ranging from −1 to +1 are investigated for different heritability values between 0.1 and 0.5. When the correlation is negative, the predicted mean sum of squares is overestimated, and this overestimation increases as the degree of correlation increases.

However, if the errors are positively associated, the expected mean sums of squares are underestimated, and they drop with an increasing degree of correlation and approach 0 as ρ tends to unity. On the other hand, when calculating the mean sums of squares due to sire, the expected mean sums of squares are underestimated when ρ is negative and overstated when the correlation is positive. As ρ tends to unity, the expected mean sum of squares due to sire approaches to its maximum value. If the correlation is positive, the heritability values are overestimated. A similar pattern may be seen at all levels of heritability. As the autoregressive coefficients rise from minus one to almost one, heritability rises from zero to nearly four. In the case of AR(2), if the AR(1) value is fixed while the AR(2) values are changed, the MSE value decreases as the correlation value increases in general. Occasionally, random tendencies are observed. We discovered that specific combinations of AR(1) and AR(2) values (0, 0), (0.1, 0.1), and (0.1, 0.5) provide superior estimates of heritability. As sample sizes are increased, the MSE values are observed to decrease. Biased estimations are obtained in practically all scenarios. Estimates based only on sire components are superior to estimates based solely on dam components. Biased estimations are obtained in virtually all circumstances. Estimates based only on sire components outperform estimates based solely on sire and dam components. In the case of AR(2), if the AR(1) value is fixed while altering the AR(2) values, the MSE value decreases as the correlation value increases in general. Occasionally, random tendencies are discovered. We discovered that combining AR(1) and AR(2) values (−0.5, −0.1) and (0.1, 0.5) yields superior estimates of heritability. As sample sizes are increased, the MSE values are observed to decrease. Biased estimates are obtained in all circumstances. Following the pattern, MSE values decline after fixing (AR(1) values increase AR(2) values up to AR(1) values 0.0). The correlation combination (−0.5,−0.1) and (0.1,0.5) produces better results than any other combination. Estimates based only on sire components outperform estimates based solely on sire and dam components. The correlation combination of (0,−0.5) and (0, 0.5) produces better results than any other combination. The MSE values drop as the sample size increases. Different correlation patterns, such as AR(1) and AR(2), can be inferred to alter heritability estimates and Means Square Error values. Various combinations are proposed for different circumstances to achieve better results.

## 4. Conclusions

In many plant and animal breeding experiments, it is seen that the observations are not independently distributed and that some type of correlation exists between the observations. In the presence of correlated error structures, the classical assumption of independence between observations is violated. Generally, plant and animal breeders are particularly interested to study the genetic components and the underlying causes of several traits. Thus, evaluating various genetic variations and inferring their inheritance based on estimations of various genetic characteristics is crucial from the perspective of plant and animal breeding programs. In the case of the full-sib model, theoretical derivation of Expected Mean sum square (EMS), considering AR(1) structure has been obtained. A numerical explanation is provided for the derived EMS considering AR(1) structure. MSE appears to be growing in tandem with the increasing trend in the correlation coefficient. It has been discovered that the MSE value increases in tandem with increasing correlation. For combined AR(1) and AR(2) structure, AR (1) is kept fixed, and AR(2) is updated. The trend in MSE behaves the same. When AR(1) and AR(2) are both adjusted, a decent correlation structure combination is found that reduces MSE. According to a simulation study, estimating heritability using only the sire component produced better results than estimating heritability using both the dam and sire components and using only the dam component.

## Figures and Tables

**Table 1:** Analysis of full-sib Families.

Source	*d.f.	*MS	*E(M.S.)
Between Sires	s-1	MSs	$\sigma_{e}^{2}+d\sigma_{d}^{2}+n\sigma_{s}^{2}$
Between dams/sires	s(d-1)	MSd	$\sigma_{e}^{2}+d\sigma_{d}^{2}$
Within Sires	sd(n-1)	MSe	$\sigma_{e}^{2}$

*d.f. = Degrees of freedom, *MS = Mean square of error, *E(MS) = Expected Mean square.

**Table 2:** Full-sib estimate of heritability and MSE values in case of correlated errors (AR(1)) and different sample sizes for the parametric value of heritability 0.10.

		Methods							
r	Sample Size	ANOVA		ML		REML		MIVQUE	
		P = 500	P = 100	P = 500	P = 100	P = 500	P = 100	P = 500	P = 100
−1	MSE $h_{s}^{2}$	0.098271 −0.181885	0.0983139 −0.183327	0.0152324 0.0014007	0.0153534 0.0002801	0.0152362 0.0014926	0.0153524 0.0003057	0.0152362 0.0014926	0.0153524 0.0003057
	MSE $h_{d}^{2}$	0.9578841 −0.383885	0.9598772 −0.383327	0.6379551 0.0015007	0.639591 0.0002901	0.6378347 0.0914926	0.6395554 0.0013057	0.6378347 0.001526	0.6395554 0.0023057
	MSE $h_{s+d}^{2}$	1.1207918 −0.28 8885	1.1210013 −0.283327	0.76337 0.0015307	0.765174 0.0003001	0.7632358 0.0024926	0.7651345 0.0004057	0.7632358 0.0014896	0.7651345 0.0013057
−0.7	MSE $h_{s}^{2}$	0.0379526 0.2647565	0.0389461 0.255177	0.0305571 0.2545453	0.0314585 0.2460257	0.0378693 0.2650561	0.0345774 0.2563335	0.0338693 0.2650561	0.0345774 0.2563335
	MSE $h_{d}^{2}$	0.3046345 0.3947565	0.3145789 0.295177	0.3150439 0.2545453	0.3234633 0.2460257	0.3041462 0.2775056	0.3126467 0.2563335	0.3111462 0.3650561	0.3126467 0.2563335
	MSE $h_{s+d}^{2}$	0.390546 0.28257565	0.4019273 0..281551	0.4024871 0.2545453	0.4121844 0.2460257	0.3900127 0.2850567	0.3998217 0.2563335	0.3900127 0.2695561	0.3998217 0.2563335
−0.5	MSE $h_{s}^{2}$	0.1491833 0.5039313	0.1595625 0.4903359	0.1488045 0.4906877	0.1495852 0.4773262	0.1691833 0.5039313	0.1595402 0.4904137	0.1591833 0.5039313	0.1595402 0.4904137
	MSE $h_{d}^{2}$	0.1125158 0.6037113	0.121275 0.5903359	0.1200414 0.5906877	0.128886 0.4773262	0.1125158 0.5139314	0.1211475 0.4904137	0.1125158 0.5989313	0.1211475 0.5904137
	MSE $h_{s+d}^{2}$	0.1625511 0.5145313	0.1733496 0.5090335	0.1720633 0.5986877	0.1829121 0.4773262	0.1625511 0.5239317	0.1732105 0.4904137	0.1625511 0.5187313	0.1732105 0.5104137
−0.3	MSE $h_{s}^{2}$	0.3455414 0.6860825	0.9612935 1.0813578	0.3284329 0.6711132	0.9270483 1.0635749	0.3455414 0.6860825	0.9612935 1.0813578	0.3455414 0.6860825	0.3612935 1.0813578
	MSE $h_{d}^{2}$	0.0426168 0.8860825	0.1239811 1.5813578	0.0457459 0.6811132	0.1137773 2.0635882	0.0426168 0.9863581	0.1239811 2.0813578	0.0426168 0.6860825	0.1239811 2.0814579
	MSE $h_{s+d}^{2}$	0.0653294 0.8860825	0.0874024 1.0983578	0.0707039 0.6921132	0.0798661 1.5635231	0.0653294 0.7860831	0.0874024 1.6781359	0.0653294 0.6860825	0.0874024 1.3893576
0	MSE $h_{s}^{2}$	0.6271941 0.893568	0.603396 0.8764351	0.6014082 0.8769554	0.5783213 0.8599561	0.6271941 0.893568	0.603396 0.8764351	0.6271941 0.893478	0.603396 0.8764351
	MSE $h_{d}^{2}$	0.043762 0.983568	0.0431264 1.8014351	0.0404352 0.8769554	0.0403303 1.8599332	0.043762 1.993535	0.0431264 1.8764345	0.043062 1.803216	0.0431264 1.8764321
	MSE $h_{s+d}^{2}$	0.0353518 0.895568	0.0372861 0.9884351	0.0345169 0.8769554	0.0369619 0.9599542	0.0353518 1.043569	0.0372861 0.9964323	0.035351 0.993568	0.0372861 0.9764363
0.3	MSE $h_{s}^{2}$	0.9853297 1.096046	0.9578041 1.0798574	0.9504265 1.0781632	0.9236276 1.0620805	0.9853297 1.096046	0.9578041 1.0798574	0.9453297 1.096046	0.9578041 1.0798574
	MSE $h_{d}^{2}$	0.1281597 2.096046	0.1225202 1.1798574	0.117433 1.0791632	0.1123769 2.0620836	0.1281597 1.106046	0.1225202 1.0798574	0.1201597 1.066046	0.1225202 1.0798574
	MSE $h_{s+d}^{2}$	0.0893778 1.196046	0.0861666 1.1985741	0.0813335 1.07781632	0.0786898 1.5627685	0.0893778 1.119604	0.0861666 1.0798574	0.0853778 1.096046	0.0861666 1.0798574
0.5	MSE $h_{s}^{2}$	1.4527074 1.310457	1.4237997 1.2956129	1.4079084 1.2915827	1.379705 1.2768211	1.4527074 1.310457	1.4237997 1.2956129	1.4227074 1.310457	1.4237997 1.2956129
	MSE $h_{d}^{2}$	0.3056672 1.810457	0.2968278 1.3956129	0.286385 1.2934827	0.2781384 2.2768342	0.3056672 2.315468	0.2968278 1.2956129	0.3056672 2.317894	0.3168278 1.2956129
	MSE $h_{s+d}^{2}$	0.2347236 1.420457	0.2281108 1.2985129	0.2182726 1.2978827	0.2122402 1.8768215	0.2347236 1.587567	0.2281108 1.2956129	0.2347236 1.657892	0.2381108 1.2956129
0.7	MSE $h_{s}^{2}$	2.5966888 1.7202475	2.5703997 1.7088652	2.5339041 1.7004875	2.5082108 1.6891521	2.5966888 1.7202475	2.5703997 1.7088652	2.5466888 1.7202475	2.5703997 1.7088652
	MSE $h_{d}^{2}$	0.8956373 2.1202475	0.8847362 1.7288652	0.8595667 1.7004875	0.8491983 2.6891534	0.8956373 2.7202475	0.8847362 1.7088652	0.8756373 2.7292471	0.8847362 2.7088652
	MSE $h_{s+d}^{2}$	0.7632251 1.8902475	0.7540315 1.7788652	0.7301186 1.7004875	0.7214505 1.9891567	0.7632251 1.8802462	0.7540315 1.7088652	0.7432251 1.8892476	0.7540315 1.7988865
1	MSE $h_{s}^{2}$	6.7818181 2.7242263	6.8135921 2.78902	6.6974755 2.7078968	6.7295237 2.7127551	6.7818181 2.7242263	6.8135921 2.72902	6.7818181 2.7242263	6.8135921 2.72902
	MSE $h_{d}^{2}$	3.7234494 2.9842263	3.7487426 2.9810223	3.6611832 2.8178968	3.6866634 3.7127583	3.7234494 3.7242973	3.7487426 3.756935	3.7234494 3.7242289	3.7487426 3.72673

r: Correlation coefficient; ANOVA: Analysis of Variance; ML: Maximum Likelihood; REML: Restricted Maximum Likelihood; MINQUE: Minimum Norm Quadratic Unbiased Estimation; MSE: Mean Squared Error; $h_{s}^{2}$: Heritability of sire component; $h_{d}^{2}$: Heritability of dam component; $h_{s+d}^{2}$: Heritability of sire and dam component; P = Sample size.

**Table 3:** Full-sib estimate of heritability and MSE values in case of correlated errors (AR (1)) and different sample sizes for parametric value of heritability 0.50.

Methods									
r	Sample Size	ANOVA		ML		REML		MIVQUE	
		P = 500	P = 100	P = 500	P = 100	P = 500	P = 100	P = 500	P = 100
−1	MSE $h_{s}^{2}$	0.4745049 0.072369	0.4821838 0.078842	0.3576657 0.0136716	0.3628984 0.0084092	0.3563739 0.0149473	0.3615089 0.0096753	0.3563739 0.0149473	0.3615089 0.0096753
	MSE $h_{d}^{2}$	0.7696736 0.082369	0.7798103 0.088842	0.6201683 0.0336716	0.6273989 0.0184092	0.6183922 0.0299473	0.6255288 0.0196754	0.6183987 0.2149465	0.6255288 0.0296765
	MSE $h_{s+d}^{2}$	1.684612 0.080469	1.7002153 0.098842	1.4624506 0.0134716	1.4741249 0.0086092	1.4595972 0.0178647	1.4711857 0.0099975	1.4595972 0.0199478	1.4711857 0.0099798
−0.7	MSE $h_{s}^{2}$	0.0362448 0.5215111	0.0402199 0.501621	0.038248 0.5081017	0.0426125 0.4884681	0.0362448 0.5215111	0.0401717 0.5016599	0.0362448 0.5215111	0.0401717 0.5016599
	MSE $h_{d}^{2}$	0.1059406 0.5675111	0.1174672 0.541621	0.1130348 0.5381017	0.1248533 0.4984681	0.1059406 0.9265113	0.1174041 0.9501659	0.1059406 1.5215784	0.1174041 1.5018753
	MSE $h_{s+d}^{2}$	0.5193806 0.5215111	0.5477033 0.531621	0.5377983 0.5181017	0.5661963 0.4804681	0.5193806 0.6289117	0.5476073 0.5516598	0.5193806 0.5900151	0.5476073 0.6016556
−0.5	MSE $h_{s}^{2}$	0.0754066 0.8097071	0.0878031 0.7851935	0.068745 0.7937283	0.0619276 0.7694161	0.0754066 0.8097071	0.0678031 0.7851935	0.0754066 0.8097071	0.0678031 0.7851935
	MSE $h_{d}^{2}$	0.0356855 0.8397071	0.0373889 0.7951935	0.0350905 0.8937283	0.0375035 0.794161	0.0356855 0.8697071	0.0373889 1.1125193	0.0356855 1.6797895	0.0373889 1.4851989
	MSE $h_{s+d}^{2}$	0.20576 0.8497071	0.2281638 0.7871935	0.2186582 0.8537283	0.2416015 0.8694161	0.20576 0.8597071	0.2281638 0.7981934	0.20576 0.895432	0.2281638 0.8964321
−0.3	MSE $h_{s}^{2}$	0.2017277 1.0127343	0.1839696 0.986424	0.1875438 0.9953401	0.170756 0.9692075	0.2017277 1.0127343	0.1839696 0.986424	0.2017277 1.0127343	0.1839696 0.986424
	MSE $h_{d}^{2}$	0.0849251 1.043343	0.077156 1.786424	0.0773452 0.9953401	0.0704789 0.9692075	0.0849251 2.0127363	0.077156 1.999427	0.0849251 2.0127376	0.077156 1.186421
	MSE $h_{s+d}^{2}$	0.0835544 1.0537343	0.0980029 0.9996424	0.0906629 0.9953401	0.1058641 0.9692075	0.0835544 1.3127347	0.0980029 0.9964398	0.0835544 1.0887344	0.0980029 0.999421
0	MSE $h_{s}^{2}$	0.4109657 1.2165893	0.3847571 1.191063	0.3884926 1.1981041	0.3633111 1.1727144	0.4109657 1.2165893	0.3847571 1.191063	0.4109657 1.2165893	0.3847571 1.191063
	MSE $h_{d}^{2}$	0.2167674 1.3165812	0.2002501 2.191063	0.2013123 1.1981041	0.1857704 1.1727144	0.2167674 2.2165887	0.2002501 2.191026	0.2167674 2.2165894	0.2002501 2.191066
	MSE $h_{s+d}^{2}$	0.0432525 1.4165833	0.0482907 1.291063	0.0434071 1.1981041	0.0493054 1.1727144	0.0432525 1.3465875	0.0482907 1.401069	0.0432525 1.3965896	0.0482907 1.291061
0.3	MSE $h_{s}^{2}$	0.6359402 1.3780468	0.6090954 1.3566075	0.6066396 1.3589356	0.5806974 1.3375917	0.6359402 1.3780468	0.6090954 1.3566075	0.6359402 1.3780468	0.6090954 1.3566075
	MSE $h_{d}^{2}$	0.3804428 1.4280464	0.3617376 2.3566075	0.358398 1.3589356	0.3405591 1.3375917	0.3804428 2.3780445	0.3617376 2.3566075	0.3804428 2.3786732	0.3617376 2.4560985
	MSE $h_{s+d}^{2}$	0.070586 1.4480321	0.0699851 1.4566075	0.0646795 1.3589356	0.0648645 1.3375917	0.070586 1.4380473	0.0699851 1.4076607	0.070586 1.978046	0.0699851 1.4568347
0.5	MSE $h_{s}^{2}$	0.910936 1.5387524	0.8871555 1.5216589	0.8748306 1.5192238	0.8518017 1.5021967	0.910936 1.5387524	0.8871555 1.5216589	0.910936 1.5387524	0.8871555 1.5216589
	MSE $h_{d}^{2}$	0.5944249 1.6387524	0.5771342 2.5216545	0.5657337 2.5192238	0.5491693 2.5021967	0.5944249 2.5387524	0.5771342 2.5213583	0.5944249 2.5387556	0.5771342 2.5216554
	MSE $h_{s+d}^{2}$	0.1488611 1.5688752	0.1460049 1.5983481	0.1366608 1.6192238	0.1344748 1.6421967	0.1488611 1.6587524	0.1460049 1.8212567	0.1488611 1.7687528	0.1460049 1.7216521
0.7	MSE $h_{s}^{2}$	1.6325969 1.8683506	1.6172951 1.8580999	1.5833143 1.848602	1.5685335 1.8383872	1.6325969 1.8683506	1.6172951 1.8580999	1.6325969 1.8683506	1.6172951 1.8580999
	MSE $h_{d}^{2}$	1.1909502 1.8883506	1.1795402 2.8580912	1.1491653 2.848602	1.1382628 2.8383872	1.1909502 2.8683513	1.1795402 2.8580999	1.1909502 2.8683507	1.1795402 2.8580991
	MSE $h_{s+d}^{2}$	0.4670591 1.8553506	0.4643053 1.8980991	0.4419508 1.899863	0.4396741 1.8999872	0.4670591 1.8989508	0.4643053 1.8980999	0.4670591 1.8983502	0.4643053 1.8980993
1	MSE $h_{s}^{2}$	4.612573 2.7529176	4.6506737 2.7603397	4.5441372 2.7368118	4.5824519 2.744318	4.612573 2.7529176	4.6506737 2.7603397	4.612573 2.7529176	4.6506737 2.7603397
	MSE $h_{d}^{2}$	3.8350907 2.9529176	3.8703735 3.7603366	3.7727696 3.7368116	3.8082345 2.744318	3.8350907 3.7529112	3.8703735 3.7603345	3.8350907 3.7529171	3.8703735 3.7603332
	MSE $h_{s+d}^{2}$	2.3642319 2.8529176	2.3932471 2.7897323	2.3155112 2.7868112	2.3446376 2.744318	2.3642319 2.9529178	2.3932471 2.9803332	2.3642319 2.9529173	2.3932471 2.8903393

r: Correlation coefficient; ANOVA: Analysis of Variance; ML: Maximum Likelihood; REML: Restricted Maximum Likelihood; MINQUE: Minimum Norm Quadratic Unbiased Estimation; MSE: Mean Squared Error; $h_{s}^{2}$: Heritability of sire component; $h_{d}^{2}$: Heritability of dam component; $h_{s+d}^{2}$: Heritability of sire and dam component, P = Sample size.

**Table 4:** Full-sib estimate of heritability and MSE values in case of correlated errors (AR(2)) and different sample sizes in case heritability of 0.10.

		Methods							
		ANOVA		ML		REML		MIVQUE	
	Sample Size	P = 500	P = 100	P = 100	P = 500	P = 500	P = 100	P = 500	P = 100
Rc1 = −1 Rc2 = −1	MSE1 $h_{s}^{2}$	0.159 −0.247	0.157 −0.231	0.015 0.005	0.014 0.009	0.015 0.010	0.013 0.017	0.018 0.017	0.017 0.015
	MSE2 $h_{d}^{2}$	17.018 4.247	16.899 4.231	14.989 3.996	14.956 3.991	14.945 3.989	14.895 3.983	14.895 3.983	14.895 3.983
	MSE3 $h_{s+d}^{2}$	3.519 2.5	3.324 2.4	3.654 2.6	3.519 2.7	3.543 2.8	3.512 2.4	3.588 2.6	3.467 2.2
Rc1 = −1 Rc2 = −0.5	MSE1 $h_{s}^{2}$	0.122 −0.168	0.120 −0.151	0.015 0.014	0.0169 0.025	0.016 0.029	0.021 0.044	0.021 0.044	0.021 0.044
	MSE2 $h_{d}^{2}$	16.392 4.168	16.273 4.151	14.915 3.986	14.835 3.975	14.809 3.972	14.698 3.956	14.698 3.956	14.697 3.956
	MSE3 $h_{s+d}^{2}$	3.519 2.7	3.123 2.6	3.413 2.5	3.351 2.6	3.567 2.7	3.534 2.2	3.619 2.5	3.519 2.3
Rc1 = 0 Rc2 = −0.5	MSE1 $h_{s}^{2}$	0.085 0.049	0.085 0.049	0.024 0.087	0.022 0.082	0.042 0.138	0.042 0.138	0.042 0.138	0.042 0.138
	MSE2 $h_{d}^{2}$	14.723 3.951	14.722 3.942	14.379 3.912	14.379 3.913	14.014 3.862	14.012 3.762	14.014 3.866	14.011 3.862
	MSE3 $h_{s+d}^{2}$	3.519 2.5	3.319 2.3	3.534 2.8	3.519 2.6	3.546 2.5	3.522 2.1	3.523 2.8	3.509 2.6
Rc1 = 0 Rc2 = 0.5	MSE1 $h_{s}^{2}$	0.104 0.103	0.103 0.103	0.038 0.117	0.037 0.114	0.064 0.181	0.063 0.180	0.064 0.181	0.062 0.179
	MSE2 $h_{d}^{2}$	14.339 3.897	14.335 3.892	14.169 3.883	14.166 3.881	13.717 3.819	13.713 3.812	13.715 3.819	13.712 3.813
	MSE3 $h_{s+d}^{2}$	3.519 2.4	3.565 2.2	3.654 2,3	3.542 2.1	3.519 2.4	3.322 2.2	3.632 2.8	3.519 2.6
Rc1 = 1 Rc2 = 0.5	MSE1 $h_{s}^{2}$	2.996 1.674	2.357 1.783	2.387 1.484	2.225 1.340	2.995 1.677	2.357 1.434	3.357 1.784	3.257 1.674
	MSE2 $h_{d}^{2}$	5.444 2.326	4.989 2.217	6.261 2.516	5.803 2.410	5.419 2.323	4.975 2.216	4.975 2.245	4.655 2.216
	MSE3 $h_{s+d}^{2}$	3.519 2.6	3.521 2.5	3.996 2.4	3.987 2.1	3.874 2.5	3.765 2.3	3.519 2.8	3.432 2.5

ANOVA: Analysis of Variance; ML: Maximum Likelihood; REML: Restricted Maximum Likelihood; MINQUE: Minimum Norm Quadratic Unbiased Estimation; MSE: Mean Squared Error; $h_{s}^{2}$: Heritability of only sire component; $h_{d}^{2}$: Heritability of only dam component; $h_{s+d}^{2}$: Heritability of both sire and dam component; MSE1, MSE2, and MSE3: MSE of only sire component, only dam component and both sire and dam component respectively. Rc1 = AR(1) and Rc2 = AR(2); P = Sample size.

**Table 5:** Full-sib estimate of heritability and MSE values in case of correlated errors (AR(2)) and different sample sizes in case heritability of 0.5.

		Methods							
		ANOVA		ML		REML		MIVQUE	
	Sample Size	P = 100	P = 500	P = 100	P = 500	P = 100	P = 500	P = 100	P = 500
Rc1 = −1 Rc2 = −1	MSE1	0.4721	0.5050	0.3202	0.3162	0.3003	0.2968	0.3003	0.2968
	$h_{s}^{2}$	−0.0169	−0.0185	0.0650	0.0801	0.1002	0.1201	0.1002	0.1201
	MSE2	11.6848	11.7266	11.0776	10.9896	10.8617	10.7475	10.8617	10.7475
	$h_{d}^{2}$	4.0169	4.0185	3.9350	3.9199	3.8998	3.8799	3.8998	3.8799
	MSE3	1.9316	1.9316	1.9316	1.9316	1.9316	1.9316	1.9316	1.9316
	$h_{s+d}^{2}$	2.0000	2.0000	2.0000	2.0000	2.0000	2.0000	2.0000	2.0000
Rc1 = −1 Rc2 = −0.5	MSE1	0.3453	0.3923	0.2758	0.2771	0.2509	0.2592	0.2509	0.2592
	$h_{s}^{2}$	0.1352	0.1244	0.1379	0.1522	0.2004	0.2144	0.2004	0.2144
	MSE2	10.713	10.8194	10.628	10.5494	10.2557	10.186	10.256	10.186
	$h_{d}^{2}$	3.8648	3.8756	3.8621	3.8478	3.7996	3.7856	3.7996	3.7856
	MSE3	1.9316	1.9316	1.9316	1.9316	1.9316	1.9316	1.9316	1.9316
	$h_{s+d}^{2}$	2.0000	2.0000	2.0000	2.0000	2.0000	2.0000	2.0000	2.0000
Rc1 = 0 Rc2 = −0.5	MSE1	0.2096	0.2908	0.1715	0.2235	0.1859	0.2490	0.1859	0.2490
	$h_{s}^{2}$	0.0492	0.5069	0.0871	0.4161	0.1381	0.5358	0.1381	0.5358
	MSE2	8.3297	8.5912	8.9155	9.0288	8.2115	8.3888	8.2115	8.3888
	$h_{d}^{2}$	3.9508	3.4931	3.9129	3.5839	3.8619	3.4642	3.8619	3.4642
	MSE3	1.9316	1.9316	1.9316	1.9316	1.9316	1.9316	1.9316	1.9316
	$h_{s+d}^{2}$	2.0000	2.0000	2.0000	2.0000	2.0000	2.0000	2.0000	2.0000
Rc1 = 0 Rc2 = 0.5	MSE1	0.2234	0.2985	0.1762	0.2259	0.2030	0.2633	0.2030	0.2633
	$h_{s}^{2}$	0.5394	0.5610	0.4271	0.4595	0.5564	0.5854	0.5564	0.5854
	MSE2	8.0183	8.2985	8.6523	8.7898	7.9191	8.1274	7.9191	8.1274
	$h_{d}^{2}$	3.4606	3.4390	3.5729	3.5405	3.4436	3.4146	3.4436	3.4146
	MSE3	1.9316	1.9316	1.9316	1.9316	1.9316	1.9316	1.9316	1.9316
	$h_{s+d}^{2}$	2.0000	2.0000	2.0000	2.0000	2.0000	2.0000	2.0000	2.0000
Rc1 = 1 Rc2 = 0.5	MSE1	0.2234	2.2715	0.1762	1.7873	0.2030	2.2703	0.2030	2.2703
	$h_{s}^{2}$	0.5979	1.9059	0.4753	1.7112	0.6121	1.9068	0.6121	1.9068
	MSE2	8.0183	2.7949	8.6523	3.3928	7.9191	2.7882	7.9191	2.7882
	$h_{d}^{2}$	3.4021	2.0941	3.5247	2.2888	3.3879	2.0932	3.3879	2.0932
	MSE3	1.9316	1.9316	1.9316	1.9316	1.9316	1.9316	1.9316	1.9316
	$h_{s+d}^{2}$	2.0000	2.0000	2.0000	2.0000	2.0000	2.0000	2.0000	2.0000

ANOVA: Analysis of Variance; ML: Maximum Likelihood; REML: Restricted Maximum Likelihood; MINQUE: Minimum Norm Quadratic Unbiased Estimation; MSE: Mean Squared Error; $h_{s}^{2}$: Heritability of only sire component; $h_{d}^{2}$: Heritability of only dam component; $h_{s+d}^{2}$: Heritability of both sire and dam component; MSE1, MSE2, and MSE3: MSE of only sire component, only dam component and both sire and dam component respectively. Rc1 = AR(1) and Rc2 = AR(2); P = Sample size.

**Table 6:** Expected mean sum of squares when errors are correlated by AR(1) and corresponding heritability values by full-sib method for different levels auto regressive coefficients for parametric heritability value 0.10.

r	Q	E(MSE)	E(MSB)	E(MSA)	$h_{s}^{2}$	$h_{d}^{2}$	$h_{s+d}^{2}$
−1	0	0	1.28	1.7309	0.154135	3.845865	2
−0.9	0.52631	0.078947	1.358947	1.809847	0.118183	2.948807	1.533495
−0.8	0.55556	0.083334	1.363334	1.814234	0.11667	2.91107	1.51387
−0.7	0.58824	0.088236	1.368236	1.819136	0.115026	2.870035	1.49253
−0.6	0.625	0.09375	1.37375	1.82465	0.11323	2.825237	1.469233
−0.5	0.66667	0.100001	1.380001	1.830901	0.111262	2.776117	1.443689
−0.4	0.71429	0.107144	1.387144	1.838044	0.109094	2.722034	1.415564
−0.3	0.76923	0.115385	1.395385	1.846285	0.106696	2.662198	1.384447
−0.2	0.83333	0.125	1.405	1.8559	0.104028	2.595628	1.349828
−0.1	0.90909	0.136364	1.416364	1.867264	0.101042	2.521118	1.31108
0	1	0.15	1.43	1.8809	0.100012	2.437167	1.267422
0.1	1.11111	0.166667	1.446667	1.897567	0.093857	2.341857	1.217857
0.2	1.25	0.1875	1.4675	1.9184	0.089483	2.232712	1.161098
0.3	1.42857	0.214286	1.494286	1.945186	0.084424	2.106489	1.095457
0.4	1.66667	0.250001	1.530001	1.980901	0.078506	1.958832	1.018669
0.5	2	0.3	1.58	2.0309	0.071491	1.783786	0.927638
0.6	2.5	0.375	1.655	2.1059	0.063041	1.572941	0.817991
0.7	3.33333	0.5	1.78	2.2309	0.052665	1.314068	0.683367
0.8	5	0.75	2.03	2.4809	0.039623	0.988646	0.514135
0.9	9.99997	1.499996	2.779996	3.230896	0.022734	0.567233	0.294983
1	122	18.3	19.58	20.0309	0.002155	0.053776	0.027965

r: Correlation coefficient; E(MSE): Expected mean square within sire; E(MSB): Expected Mean square between dams within sire; E(MSA): Expected Mean square between sire; MSE: Mean Squared Error; $h_{s}^{2}$: Heritability of only sire component; $h_{d}^{2}$: Heritability of only dam component; $h_{s+d}^{2}$: Heritability of sire and dam component; Q = Polynomial of correlation coefficient of order $n_{ij-1}$

## Data Availability

Not applicable.
